# A Comprehensive Review on Metal Catalysts for the Production of Cyclopentanone Derivatives from Furfural and HMF

**DOI:** 10.3390/molecules28145397

**Published:** 2023-07-14

**Authors:** Ying Duan, Yiyi Cheng, Zhi Hu, Chenxu Wang, Dong Sui, Yanliang Yang, Tianliang Lu

**Affiliations:** 1 Henan Key Laboratory of Function-Oriented Porous Materials, College of Chemistry and Chemical Engineering, Luoyang Normal University, Luoyang 471934, China; duanying@mail.ustc.edu.cn (Y.D.); 19588517002@163.com (C.W.); suidonghy@mail.nankai.edu.cn (D.S.); 2 College of Food and Drug, Luoyang Normal University, Luoyang 471934, China; 3 School of Chemical Engineering, Zhengzhou University, Zhengzhou 450001, China; 18816706408@163.com (Y.C.); wmhzuuu@163.com (Z.H.)

**Keywords:** furfural, HMF, cyclopentanone derives, hydrogenation, metal catalyst

## Abstract

The catalytic transformation of biomass-based furan compounds (furfural and HMF) for the synthesis of organic chemicals is one of the important ways to utilize renewable biomass resources. Among the numerous high-value products, cyclopentanone derivatives are a kind of valuable compound obtained by the hydrogenation rearrangement of furfural and HMF in the aqueous phase of metal–hydrogen catalysis. Following the vast application of cyclopentanone derivatives, this reaction has attracted wide attention since its discovery, and a large number of catalytic systems have been reported to be effective in this transformation. Among them, the design and synthesis of metal catalysts are at the core of the reaction. This review briefly introduces the application of cyclopentanone derivatives, the transformation mechanism, and the pathway of biomass-based furan compounds for the synthesis of cyclopentanone derivatives. The important progress of metal catalysts in the reaction since the first report in 2012 up to now is emphasized, the characteristics and catalytic performance of different metal catalysts are introduced, and the critical role of metal catalysts in the reaction is discussed. Finally, the future development of this transformation process was prospected.

## 1. Introduction

The large-scale application of non-renewable fossil resources has caused various problems while promoting tremendous social development. Resource shortages, excessive carbon emissions, and environmental pollution are typical problems faced by modern society. So, people are forced to seek cleaner energy and resources with zero carbon emissions. There are various types of clean energy, such as solar energy, wind energy, geothermal energy, and hydropower, while biomass is considered an organic carbon resource with zero carbon emissions. As an organic carbon resource that can be extensively regenerated through photosynthesis from CO_2_ in the air every year, biomass is an ideal source of resources for human demand [1,2,3].

Biomass is mainly composed of cellulose, hemicellulose, and lignocellulose. Cellulose is a macromolecular polysaccharide composed of glucose as the only structural unit. Hemicellulose is a heterogeneous polymer composed of several different types of monosaccharides, including pentose and hexose. Lignin is a complex phenolic polymer formed from phenolic monomers. After careful analysis of the main components of biomass, carbohydrates were found to be the main components. Thus, the conversion of carbohydrates has become an important topic for biomass conversion [4,5,6,7].

It is not easy to obtain valuable organics from carbohydrates directly, considering the complexity of biomass carbohydrate compounds. Thus, platform compounds are usually used to bridge the gap between organic chemicals and complex biomass molecules. Biomass-derived furans, furfural and 5-hydroxymethylfurfural (HMF), can be readily obtained from carbohydrates by catalytic dehydration and be converted into a variety of high-value-added compounds [8,9,10,11,12,13,14,15]. So, biomass-derived furans are viewed as bridges for carbohydrate compounds.

**Figure 1:**
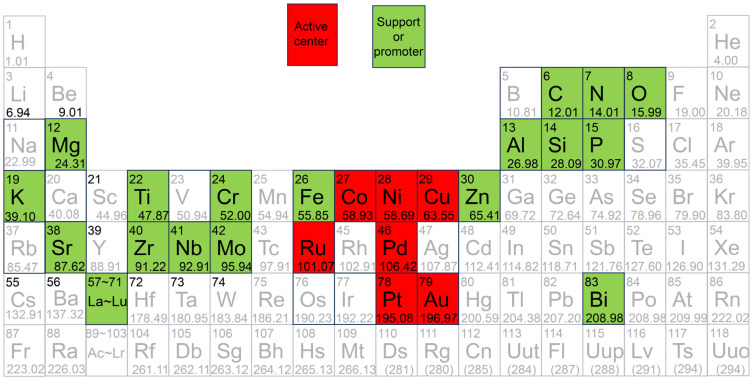
Chemical elements involved in the catalysts in the transformation of furfurals to cyclopentanone derives.

The route for the transformation of carbohydrate compounds to chemicals has been widely studied. Carbohydrate was first converted to furfural or HMF by dehydration catalyzed by acid. Then, the furfural and HMF could be converted to various compounds through hydrogenation, oxidation, and other reactions. Generally, the products of furfural and HMF can be divided into two distinct categories. One is a product that maintains the furan structure. For example, 2,5-furandicarboxylic acid can be obtained by the oxidation of HMF and can be used as a highly promising polymer monomer [16,17,18]. The hydrogenolysis of HMF can produce 2,5-dimethylfuran, whose property is suitable for fuel, as a potential fuel and additive [19,20,21]. The other category is to change the furan ring structure in HMF and furfural to expand the product range. Chain and ring compounds can be obtained. Chain compounds are composed of levulinic acid [22,23], diols [24,25,26], polyols [27,28,29], 2,5-hexanedione [30,31,32], and 1-hydroxy-2,5-hexanedione [31,32,33,34], which are all valuable chemicals with a wide range of application. Ring compounds contain non-furan heterocycles [35,36,37,38] and carbon rings. In 2012, Hronec et al. [39] first reported the conversion of furfural to cyclopentanone (CPO), and then Ohyama et al. [40] realized the transformation of HMF to a cyclopentanone derivative, 3-hydroxymethylcyclopentanone (HCPN). Since then, the conversion of HMF and furfural to cyclopentanone derivatives has attracted much attention due to the huge application prospects of cyclopentanone derivatives [41]. In this review, we focus on the metal catalyst used in the transformation of HMF and furfural to cyclopentanone derivatives in recent years (Figure 1). We summarize the differences between the different metals in this transformation, as well as the roles of different parts of the catalyst.

## 2. Application of Cyclopentanone Derivatives

**Figure 2:**
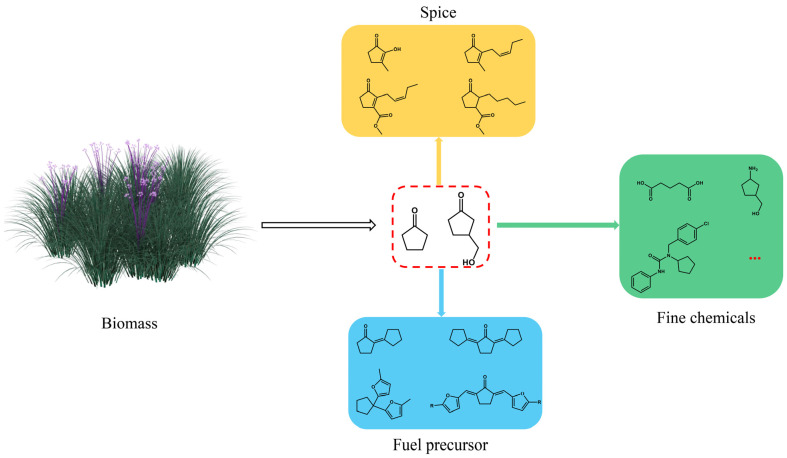
Potential application for the cyclopentanone derivatives.

**Scheme 1:**
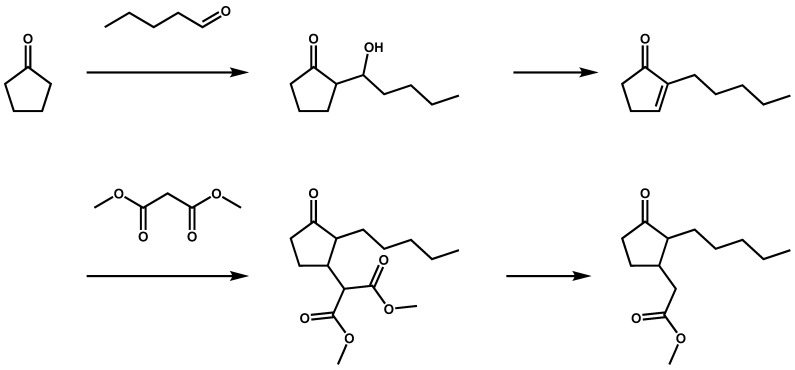
Reaction pathway for production of methyl 2,4-dihydroxybenzoate from CPO.

**Scheme 2:**
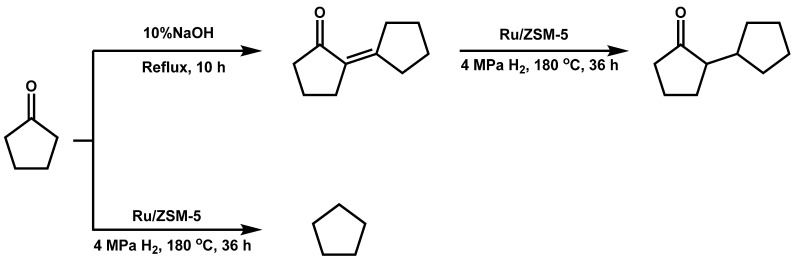
Schematic description for production of cycloalkanes. Reproduced from Ref. [57] with permission from the Royal Society of Chemistry.

CPO and its derivatives are chemicals of significant application value. CPO has been widely used as a solvent in the electronics industry due to its proper solubility for various resins. Cyclopentanone derivatives are also important intermediates in organic synthesis (Figure 2). CPO is the main structure of jasmine spices and thus can be used to synthesize a series of precious jasmine spices. For example, methyl 2,4-dihydroxybenzoate can be synthesized through the reaction route shown in Scheme 1. With four steps of reaction, methyl 2,4-dihydroxybenzoate can be obtained with high yield by using pentanal and cyclopentanone as raw materials. Methyl 2,4-dihydroxybenzoate is a widely used jasmine flavoring compound, with an annual output of more than 10,000 tons. As CPO has a similar structure to cyclohexanone, cyclopentanone can also be used to synthesize polymer monomers. Dicarboxylic acids and amine compounds can be obtained from cyclopentanone derivatives. What is more, with the development of biomass-based cyclopentanone production technology, the preparation of fuel or fuel additives has emerged as a very promising topic [42,43,44,45,46,47,48,49,50,51,52,53,54,55,56]. Through aldol condensation reaction or other reactions of carbon chain growth, CPO can be used as a substrate for the preparation of compounds with carbon chain lengths falling in the range of diesel. Then, the compounds can be subjected to hydrogenolysis and deoxygenation to produce diesel fuel. The most popular reactions are the aldol condensation of CPO itself or CPO and furfural. Xu et al. first reported the aldol condensation of CPO catalyzed by 10% NaOH to prepare [1,1′-bi(cyclopentylidene)]-2-one and then hydrogenolysis at Ru/ZSM-5 at 180 °C. 1,1′-Bi(cyclopentane) was obtained as a potential jet fuel with high selectivity (Scheme 2). The aldol condensation of CPO with furfural is also a good choice to obtain C10 and C15 fuel precursors. Another essential advantage of preparing fuel from CPO is that hydrocarbon compounds with cyclic structures have a higher density than chain compounds, which are very suitable for use as jet fuel.

## 3. Mechanism for the Production of Cyclopentanone Derivatives

**Scheme 3:**
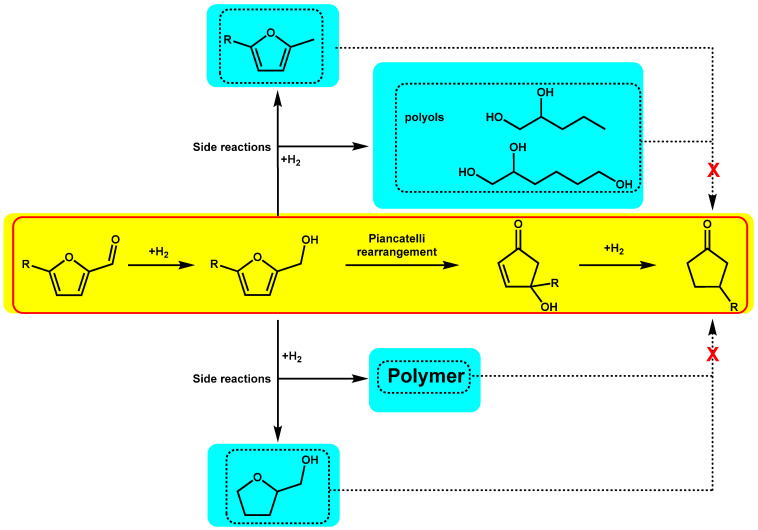
Pathway for transformation of HMF and furfural to cyclopentanone derivatives.

**Scheme 4:**
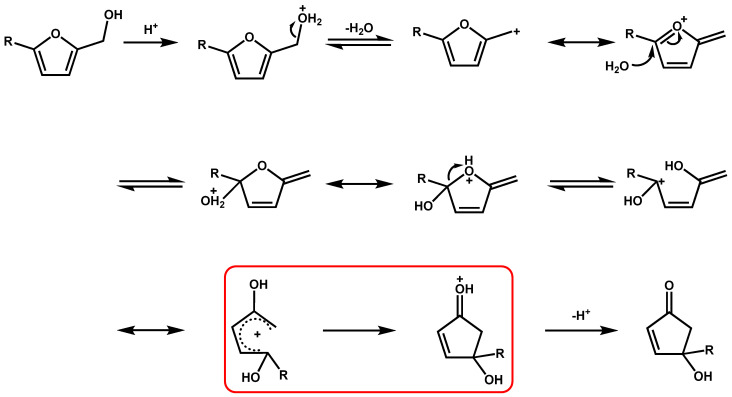
Mechanism for Piancatelli rearrangement.

The widely accepted mechanism for the conversion of furfural and HMF to cyclopentanone derivatives is shown in Scheme 3. The reaction is a multistep tandem reaction, including hydrogenation and rearrangement reactions. Furfural and HMF are first hydrogenated to furfuryl alcohol (FOL) and 2,5-bihydroxymethylfuran (BHMF), and then the Piancatelli rearrangement of FOL and BHMF realizes the transformation from the furan ring to the five-membered carbon ring. At last, the hydrogenation of Piancatelli rearrangement products generates the final product cyclopentanones or cyclopentanols. Both hydrogenation and acid active centers are needed for the reaction. The mechanism for the Piancatelli rearrangement is shown in Scheme 4 [58,59,60,61]. The reaction typically occurs in water catalyzed by acid. In the presence of an acid, the FOL or BHMF will form a 4-π-electron-conjugated system intermediate and then realize ring closing through an electrocyclic reaction. The activity of metal hydrogenation active centers needs to be finely regulated to avoid side reactions. The metal should have a higher activity of aldehyde group hydrogenation to generate alcohols while simultaneously posing the lowest possible hydrogenation activity for the furan ring and the hydrogenolysis of the C-O bond. Once the furan ring hydrogenation or the C-O bond cleavage product is generated, the products cannot further isomerize to produce cyclopentanone derivatives. Another important issue to note is the polymerization of BHMF and FOL in a hot acidic aqueous solution. Traditionally, FOL is used as a raw material for preparing resin and is very easy to undergo polymerization reactions under acidic conditions. The inhabitation for the generation of oligomers or polymers is an important topic for the production of cyclopentanone derivatives from furfural and HMF.

## 4. Reaction Conditions

In most cases, water is a necessary solvent for the production of cyclopentanone derivatives from furfural and HMF. As shown in Scheme 4, water not only plays the role of a solvent, but also participates in the reaction as a reactant. This is further confirmed by an isotope experiment. When heavy-oxygen water is used as a solvent, ^18^O is observed in CPO for the hydrogenation of furfural, and the isomerization reaction of furfuryl alcohol in ordinary water and heavy water shows secondary kinetic deuterium isotope effects. So, water should be involved in the rearrangement reaction as a reactant. Usually, pure water is used as a solvent, and mixed solvents containing water have lower selectivity for CPO (Table 1). Wang et al. studied the conversion of furfural on NiFe/SBA-15 in methanol/water mixed solvents. FOL was the main product for the pure methanol (Table 1, Entry 1), tetrahydrofurfuryl alcohol (THFA) was the main product for the methanol-dominated solvent, and CPO was the main product for the water-dominated solvent (Table 1, Entry 4). The pure water solvent gave the highest selectivity to CPO (75.6%, Table 1, Entry 5). After optimizing the conditions, the yield of CPO could reach 90%.

Temperature is another vital reaction parameter for the production of cyclopentanone derivatives from furfural and HMF. Generally, it is necessary to perform the reaction at a temperature above 120 °C for a satisfactory yield of cyclopentanone whether on noble metal or non-noble metal catalysts. According to Scheme 3, though furfural and HMF can be hydrogenated to FOL and BHMF at lower temperatures on noble metal catalysts, the rearrangement of the furan ring towards the five-membered carbon ring needs to be carried out under high-temperature conditions. So, at lower temperatures, the main products are FOL and BHMF.

The concentration of the substrate needs to be low to obtain a satisfactory yield of cyclopentanone derivatives from furfural and HMF. Most of the values fall within the range of 1.0–5.0%. As FOL is traditionally used for preparing furfuryl alcohol resin and is more unstable than furfural, it is easy to perform the polymerization reaction in hot water. So, when the concentration of reactants is increased, the sharp increase in polymerization side reactions leads to low selectivity to CPO.

In general, the reaction should be conducted under acidic conditions, as the Piancatelli rearrangement should be catalyzed by the acidic site. The weak acidity is beneficial for the generation of cyclopentanone derivatives, while the strong acidity to generate polymers. In most cases, the acidity of the bifunctional metal catalysts formed by loading metal on acidic supports adequacy. It was found that the Bronsted acidic site is beneficial to the formation of polymerization products, while Lewis acid is beneficial to the formation of cyclopentanones from the rearrangement reaction.

## 5. Production of CPO from Furfural

**Figure 3:**
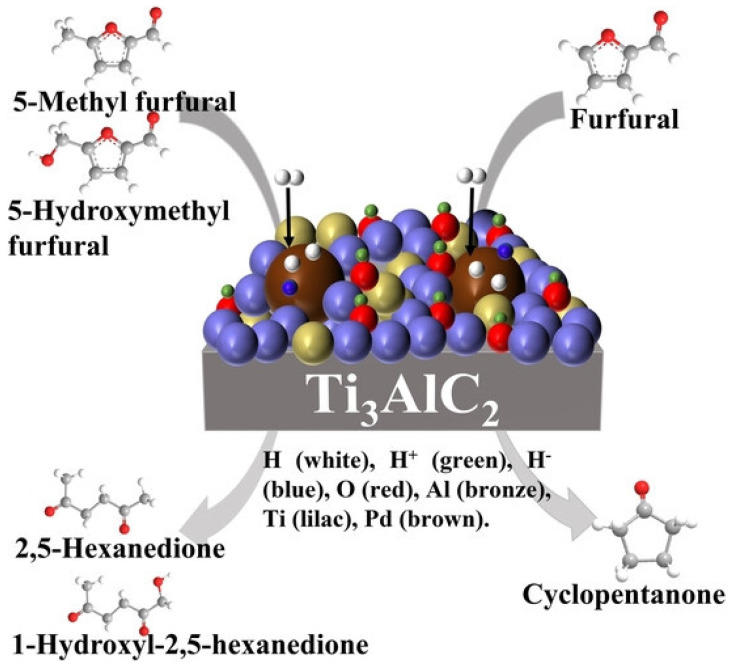
Diagrammatic sketch for Lewis H^+^-H^−^ pairs generated by in situ hydrogen spillover. Adapted with permission from Ref. [63]. Copyright 2023 John Wiley and Sons.

The primary role of metal catalysts is to provide hydrogenation active centers to catalyze the hydrogenation of furfural and HMF to FOL and BHMF for the hydrogenation of the intermediates after the rearrangement of FOL or BHMF. As the rearrangement reaction needs to be carried out under slightly acidic conditions, acid support is favored for the metal catalysts, forming a bifunctional catalyst for the production of cyclopentanone derivatives from furfural and HMF. Recently, it was found that the metal–support interaction not only affects the activity of the catalyst, but also has a significant impact on the selectivity for the supported metal catalyst. For example, in situ hydrogen spillover generates frustrated Lewis H^+^-H^−^ pairs that provide additional acid sites for the ring rearrangement of furans (Figure 3) [63]. In this section, the recent progress in the hydrogenation of furfural to CPO is summarized.

### 5.1. Single Non-Noble Metal Catalyst

#### 5.1.1. Cu

The most used non-noble metal catalyst for the hydrogenation of furfural to CPO is the Cu catalyst. Cu-based metal catalysts have low activity for hydrogenation due to the full 3D orbit. However, Cu has very high selectivity for the hydrogenation of C=O to alcohol. Cu is inert for the hydrogenolysis of C-O and the hydrogenation of the furan ring in FOL, which gives the production of methyl furan and tetrahydrofurfuryl alcohol as by-products.

Copper containing hydrotalcite is a good precursor for the preparation of a high-performance catalyst for the hydrogenation of furfural. Zhang et al. [64] prepared a series of CuZnAl catalysts for the production of CPO from furfural through the co-precipitation of Cu(NO_3_)_2_·3H_2_O, Zn(NO_3_)_2_·6H_2_O, and Al(NO_3_)_3_·9H_2_O by Na_2_CO_3_ (Table 2, Entry 1). The effects of the molar ratios for Cu/Zn and the reaction conditions were studied, and a 62% yield of CPO was obtained from furfural in water on the CuZnAl-500-0.5 catalyst. Xiao et al. [65] prepared Cu-Mg-Al hydrotalcite-derived catalysts with a (Cu + Mg)/Al mole ratio of 3 (Table 2, Entry 2). By adjusting the Cu/Mg mole ratio, 98.5% conversion of furfural and 94.8% selectivity to CPO were obtained with a Cu/Mg mole ratio of 0.2. Huang et al. [66] realized the selective conversion of furfural to FOL, CPO, or cyclopentanol (CPL) by tuning the reaction conditions using Cu_0.4_Mg_5.6_Al_2_ as the catalyst (Table 2, Entry 9). A 98.1% yield of CPO was obtained under 0.2 MPa H_2_ at 180 °C, while a 98.6% yield of CPL was achieved at 190 °C, 2.0 MPa H_2_. The effect of furfural concentration was also studied by using 15–30 wt.% furfural in water, and a 97.1% yield of CPO was obtained from 20 wt.% of furfural solution.

The interaction between metal supports has a significant impact on catalytic performance. Li et al. [67] prepared Cu/ZrO_2_ with an adjustable surface structure by the one-pot oxidation–reduction method. There was a strong interaction between Cu and ZrO_2_. A Cu^+^-O-Zr-like structure formed at the metal–support interface. Both the Cu^+^/(Cu^0^ + Cu^+^) ratio and the surface acidic site played key roles in the high selectivity to CPO. Feng et al. [68] performed the conversion of furfural to CPO or CPL on Cu/Fe_3_O_4_ prepared by the co-precipitation method. Cu and the support showed an obvious interaction, revealed by the characterization (Table 2, Entries 6 and 7). The selectivity to CPO and CPL could be monitored by the Cu content and the reaction conditions. A 91% yield of CPO was obtained on 10%Cu/Fe_3_O_4_, while an 82% yield of CPL was achieved on 50%Cu/Fe_3_O_4_. He et al. [69] produced defect-rich nano-twin Cu particles whose structure could be controlled by the gradient reduction strategy for the conversion of furfural (Table 2, Entry 14). Multilevel surface defect sites originating from planar defects are instrumental in enhancing the selectivity to CPO via the hydrodeoxygenation and hydrogenation of C-O in the intermediate of 4-hydroxy cyclopentenone to C=C. The performance of these defect-rich nano-twin Cu particles increased by 50% compared to the regular spherical Cu particles. Liang et al. [70] investigated the effect of Cu–support interfaces on the conversion of furfural to CPO or CPL (Table 2, Entry 21). Alumina-decorated Cu/ZnO was prepared through the co-precipitation method. The metal–support interaction and contacting interfaces could be adjusted by the atomic ratio between Al/Zn. The Cu-AlOx interface was conducive to generating the final product of CPL, while Cu-Zn-Ox was favored for the production of CPO.

Li et al. [71] studied the nature of polymeric condensates for the conversion of furfural to CPO using Cu-based catalysts (Table 2, Entry 22). By the combination of TG, PXRD, FT-IR, and pyrolysis GC-MS characterization, it was found that the polymer on the catalyst surface after the reaction was conjugated polymers derived from polyfurfuryl alcohol different from traditional polyfurfuryl alcohol. Furfural did not form polymers on the surface of the catalyst in the reaction condition. The introduction of alkali or alkaline earth metal could increase the carbon balance by inhibiting the generation of polymers, and Cu-Ba showed the best performance.

#### 5.1.2. Ni

Generally speaking, Ni has higher catalytic hydrogenation activity than Cu. Thus, when Ni is used for the conversion of furfural to CPO, much attention should be paid to regulating the activity of Ni to avoid the hydrogenation of the furan ring and the hydrogenolysis of carbon–oxygen bonds.

A Ni/HY catalyst for the conversion of furfural to CPO was prepared using graded HY zeolite as the support [72]. By using sodium alginate as a template for introducing mesopores, the formed graded HY proved to be a good catalyst support for preparing cyclopentanone from furfural (Table 2, Entry 3). The high specific surface area, large mesoporous structure, and suitable acidity were attributed as reasons for the high yield of CPO.

TiO_2_-supported Ni catalysts were prepared through the wet impregnation method. Multiple characterization methods proved the formation of the Ni-NiO heterojunction structure. Further, by adjusting the proportion of different crystal forms in titanium oxide, Ni-NiO/TiO_2_-Re450 proved to be an effective catalyst for the conversion of furfural to CPO [73]. An 87.4% yield of CPO was obtained, and the catalyst could be used at least five times (Table 2, Entry 10).

The conversion of furfural to CPO can be conducted in a Pickering emulsion. Appropriate mass transfer effects and the friendly catalyst separation method provide an excellent strategy to overcome the limitations of traditional phase transfer catalysis. Escalona et al. employed a carbon nanotube as the support for Ni for the preparation of Ni/CNTox. The catalyst mainly was distributed at the liquid–liquid interface in a Pickering emulsion composed of dodecane–water. A 25% yield of CPO with 35% conversion of furfural was achieved at the optimal conditions [74]. Amphiphilic CNT-supported Ni could be obtained by tuning the wettability of catalysts through different acid treatment methods (Table 2, Entry 12). The obtained Ni/CNT was used as the emulsion stabilizer composed of water and dodecane and as the catalyst for the conversion of furfural. Results showed 69% selectivity to CPO and 94% conversion of furfural in the mixed solvent (Table 2, Entry 11) [75]. The effect of Ni content was also investigated. It was found that lower Ni content was favored for the high selectivity to CPO (Table 2, Entry 18) [76].

The role of acid was studied by adding additional acidic additives using Ni/SiC with negligible surface acidity and alkalinity as the hydrogenation catalyst (Table 2, Entry 13) [77]. Both Brønsted and Lewis acids could catalyze the rearrangement of FOL, and CrCl_3_ showed the highest yield of CPO. The main by-product was THFA, which was produced by the hydrogenation of the furan ring in FOL. To avoid the excessive hydrogenation of FOL to THFA and provide active acidic centers, Ni/Al_2_O_3_ was modified by phosphorus. The formation of the AlPO_4_ phase after the introduction of phosphorus provided the acidic center on the surface of the catalyst, while the formed NixP hydrogenation active center showed the inhibition of catalytic activity for the hydrogenation of the furan ring. The combination of acid sites and the NixP hydrogenation active center gave a very high yield of CPO from furfural. The effect of the heteroatom on the transformation of furfural to CPO was further investigated by the study on the effect of the N/P co-doped nickel encapsulated in carbon (Table 2, Entry 24) [78]. By the pyrolysis of Ni-[BMIM]PF6 under N_2_, Ni@NP-C with highly dispersed Ni could be obtained, and N/P could be introduced simultaneously. The introduction of N and P not only increased the adsorption of substrates, but also introduced acidic sites that promoted furfuryl alcohol rearrangement.

Nickel phosphide has proved to be a good hydrogenation catalytic center for the conversion of furfural to CPO. An electroless plating method was used to prepare a bifunctional Ni_3_P/γ-Al_2_O_3_ catalyst. γ-Al_2_O_3_ provided a large number of weak and medium acidic sites. Together with the Ni_3_P hydrogenation center, the bifunctional Ni_3_P/γ-Al_2_O_3_ obtained a 74.6% yield of CPO from furfural (Table 2, Entry 27) [79]. Triple-functional Sr_2_P_2_O_7_/Ni_2_P was synthesized by a one-pot hydrothermal synthesis method. The introduction of Sr to the nickel phosphide greatly improved the catalytic activity of the catalyst that the reaction could perform at 0.1 MPa H_2_. Acid and alkaline active centers were also brought into the catalyst. The combined action of the hydrogenation center and the acid-base active center achieved a 96% yield of CPO from furfural under atmospheric pressure of H_2_ (Table 2, Entry 28) [80].

The excessive hydrogenation of FOL to THFA on Ni catalysts could be adjusted through the control of the number of active catalytic centers on Ni/SiO_2_ (Table 2, Entry 20) [81]. As the furan ring was a five-center six-electron-conjugated system (π_5_^6^), the hydrogenation of the furan ring required the synergistic effect of multiple catalytic centers. On the contrary, the hydrogenation of C=O needs fewer Ni centers. So, the reduction of the number of catalytic active centers was not conducive to the hydrogenation of furan rings, and the production of the by-product of THFA was inhibited. On 0.8%Ni/SiO_2_, 98.2% selectivity to CPO was achieved at 160 °C.

The conversion of furfural to CPO could be achieved in a nanoreactor. The hollow carbon ball encapsulated Ni nanoreactor (Ni@HCS) could be prepared by encapsulating metallic nickel in the hollow carbon spheres (250 nm) (Table 2, Entry 23) [82]. A 99.1% yield of CPO could be obtained from furfural. The nanoreactor could protect Ni from Ni leaching, and the activity could be maintained after 10 cycles of use.

#### 5.1.3. Co

Recently, the feasibility of utilizing a single-metal cobalt catalyst for the selective hydrogenation of furfural to produce CPO was investigated. Co typically has a very high catalytic activity such that CPO can be easily hydrogenated to CPL under reaction conditions.

Deng et al. [83] fabricated Co-N sites with doped Co@Co-NCs in which the size of Co could be turned (Table 2, Entry 25). Though there were no prominent acidic sites on the catalysts themselves, the acidic sites OH^δ−^-Co-N-H^δ+^ could be generated by the heterocracking of hydrogen and the assistance of H_2_O. The in-situ-generated acidic sites showed sufficient acidity to catalyze the rearrangement reaction. A 95.2% yield of CPL was obtained under the optimal conditions.

A carbon-nanotube-supported cobalt catalyst doped by N (CIy-T) was prepared by the pyrolysis of ZIF-67 at different temperatures (Table 2, Entry 26) [84]. The effect of the solvent on the hydrogenation of furfural on CIy-T was studied. It was found that the Brønsted acid sites were maintained when the reaction was performed in water, while the Brønsted acid sites disappeared in other solvents. Thus, the maintained Brønsted acid sites could catalyze the Piancatelli rearrangement to make CPO and CPL the main products. On the other hand, THFA was the main product when ethanol was used as a solvent.

Nb_2_O_5_-supported CoOx was prepared by the wetness impregnation method to obtain a bifunctional catalyst with a hydrogenation center and an acid site. CoOx was considered to be the key factor in the transformation. The introduction of cobalt oxide on the niobium oxide promoted the chemical adsorption of carbonyl groups and adjusted the Brønsted/Lewis acidic site ratio, which was suitable for the rearrangement reaction. Theoretical calculations proved that the reaction intermediates had poor stability on the catalytic active center and were easy to convert. So, an 82% total yield of CPL + CPO was achieved (Table 2, Entry 29) [85].

**Table 2:** Conversion of furfural to CPO on Cu, Ni and Co.

Entry	Catalyst	Concn. ^a^ (wt.%)	T (°C)	t (h)	P (MPa)	Conv. (%)	Yield ^b^ (%)	Ref.
1	CuZnAl-500-0.5	3.1	150	6	4	>90	62	[64]
2	Cu-Mg-Al	5.5	140	10	4	98.5	(93.4)	[65]
3	20wt.%Ni/HY-0.018	5.0	150	9	4	96.5	86.5	[72]
4	RANEY^®^ Ni	8.8	180	4	1 N_2_	94.4	75.4	[86]
5	Cu/ZrO_2_	3.1	150	4	1.5	100	91.3	[67]
6	10%Cu/Fe_3_O_4_	1.0	170	4	3	100	91	[68]
7	50%Cu/Fe_3_O_4_	1.0	170	3	3	100	(82)	[68]
8	Cu_0.4_Mg_5.6_Al_2_	4.6	180	5	0.2	100	98.1	[66]
9	Cu_0.4_Mg_5.6_Al_2_	4.6	190	12	2	100	(98.6)	[66]
10	Ni-NiO/TiO_2_-Re450	1.0	140	6	1	100	87.4	[73]
11	Ni/CNTox1	2.2	200	4	2	94	64.9	[75]
12	Ni/CNTox	2.2	200	1	2	35	25	[74]
13	Ni/SiC-CrCl_3_	2.3	160	2	3	99.9	88.1	[77]
14	Cu0-Zn(Al)(Zr)O-2	4.8	160	3	2.5	100	92	[69]
15	NiMo/CNT	4.8	140	2	4	94	(56.3)	[87]
16	Ni–P/γ-Al_2_O_3_	1.0	190	2	3	97.6	93.8	[88]
17	15%Ni + 10%P	1.0	150	2	3	96.7	90.1	[88]
18	Ni/CNTox	2.2	200	2		10	6.3	[76]
19	0.8%Ni/SiO_2_	1	160	2	3	34.2	33.6	[81]
20	2.0%Ni/SiO_2_	1	160	2	3	98.8	80.3	[81]
21	Cu/ZnO-Al_3_	3.7	160	4	4	>99	(60.7)	[70]
22	Cu–K	1.4	200	4	1	100	54.8	[71]
23	Ni@HCS	1	150		2	100	99.1	[82]
24	Ni@NP-C	1.9	130	2	1.5	100	86.7	[78]
25	Co@Co-NC	4.1	150	6	4	99.9	(95.1)	[83]
26	CI0.25-700	0.7	130	10	1.5	100	79.4 (20.4)	[84]
27	Ni_3_P/γ-Al_2_O_3_	4.8	180	1	4	99.9	74.6	[79]
28	(Sr_2_P_2_O_7_)0.40/Ni_2_P	1	150	2.2	0.1	-	96	[80]
29	CoOx/Nb_2_O_5_	2.0	160	6	2	100	61 (21)	[85]

^a^ The concentration was calculated based on the data assuming the density of water is 1 g·mL^−1^. ^b^ The yield was the value provided by the literature or calculated by the product of selectivity and conversion. The value in the bracket represents cyclopentanol.

### 5.2. Bimetallic Non-Noble Metal Catalyst

Monometallic catalysts (Cu, Ni, and Co) have shown good catalytic effects on the catalytic conversion of furfural to CPO. However, the inherent defects of the monometal limit the further improvement of comprehensive catalytic performance. Although Cu has high catalytic conversion selectivity, the activity for Cu is usually lower, and harsh reaction conditions (higher temperatures and pressures) are needed. On the contrary, a high-activity Ni catalyst easily leads to the excessive hydrogenation of FOL to form THFA, while the Co catalyst keeps the reaction in the CPO stage. The combination of different catalytic active centers to adjust the electronic structure through metal–metal interactions is a good way to achieve high-performance catalysts. Copper is one of the popular components for bimetallic catalysts due to its unique selectivity for carbonyl hydrogenation.

#### 5.2.1. NiCu

NiCu/SBA-15 was synthesized using a dual-solvent method to form uniformly distributed NiCu alloy nanoparticles within pores. The addition of Cu to the Ni not only improved the activity for furfural conversion compared to Ni/SBA-15, but also significantly increased the selectivity to CPO. FOL, 4-hydroxy-2-cyclopentenone, and 2-cyclopentenone were identified first as the key intermediates. The adsorption competition of the aldehyde group in furfural on the catalysts rather than FOL and the carbonyl in CPO was attributed to the high selectivity to CPO (Table 3, Entry 1) [57]. Then, Al-MCM-41 was used as the support instead of SBA-15 for NiCu. The introduction of Al to MCM-41 played the role of anchoring and guaranteeing the formation of highly dispersed Cu-Ni bimetallic nanoparticles as well as providing L-acid centers. The yield of CPO was closely related to the acidity or alkalinity of the solution and the concentration of furfural. The polymerization of the intermediates was attributed as the reason to prevent selectivity improvement (Table 3, Entry 12) [89].

Cu-Ni-Al was synthesized from hydrotalcite-like precursors prepared by the co-precipitation method. By fixing the mole ratio of (Cu + Ni) to Al as 3 while changing the mole ratio of Ni to Cu, a 95.8% yield of CPO was obtained from furfural on Cu-Ni-Al with a Cu/Ni/Al mole ratio of 1:14:5 (Table 3, Entry 2) [90].

Highly active CuNi@C could be obtained by the pyrolysis of Cu-BTC pre-adsorbing nickel ions. Compared to traditional preparation methods, high specific surface areas and smaller and more uniform nanoparticles were obtained by this method. The carbon matrix was capable of effectively dispersing and stabilizing catalytic active centers to obtain a 96.9% yield of CPO (Table 3, Entry 5) [91].

Zhang et al. prepared NiCu/SiO_2_ for the hydrogenation of furfural to CPO through an alkaline hydrothermal method with different mole ratios of Ni to Cu. The introduction of Cu increased the reducibility of Ni and weakened the chemical adsorption ability of C=C in the furan ring by forming a NiCu alloy. The presence of a large amount of silicon hydroxyl groups was confirmed through XPS and infrared spectroscopy and was the key factor for the rearrangement of FOL (Table 3, Entry 14) [92]. NiCu/SiO_2_-AE-450 with a single-layer bimetallic silicate structure enhanced the interaction between NiCu and SiO_2_ and was used for the production of CPO from furfural (Table 3, Entry 15) [93]. The effect of the addition of Cu to Ni was confirmed. The electronic structure of Ni was changed, resulting in increased interaction with carbonyl groups and weakened adsorption of the furan ring on the surface. This favored the formation of FOL as an intermediate, which was beneficial for the formation of CPO.

The different catalytic effects of Cu, Ni, and NiCu alloys were studied based on the combination of in situ spectroscopy and density functional theory calculations (Table 3, Entry 10) [94]. It was demonstrated that the different catalytic performance comes from the η1(O)-type adsorption sites for the carbonyl group on NiCu, which is different from the dominant η2(C, O) adsorption mode on monometallic Ni nanoparticles.

#### 5.2.2. Cu-Co

Cu-Co was prepared by different methods of co-precipitation and sol–gel to study the role of Cu and Co species in the conversion of furfural to CPO. Cu^0^, Co^0^, and Cu(I) were the active hydrogenation centers with different proportions using different preparation methods. Cu(I) also played the role of Lewis acid sites in the reaction. Cu-Co-CP-500 prepared by the co-precipitation method mainly showed Cu species on the surface and had weak hydrogenation activity. So, CPO was the main product on Cu-Co-CP-500. On the contrary, Co^0^ and Cu_2_O were the main active centers on Cu-Co-OG-500 prepared by the sol–gel method to make the CPL the main product (Table 3, Entries 3 and 4) [95]. The effect of cobalt on the selective transformation of furfural to CPO over a CNT-supported copper-based catalyst was studied (Table 3, Entry 16) [96]. The addition of Co to Cu both increased the activity for furfural, but the selectivity to CPO was also increased as well. Zhao et al. (Table 3, Entry 7) [97] reported a highly active catalyst composed of Cu, Co, and three-dimensional porous carbon as the support using glucose as the carbon source. There were interactions and synergistic effects between the two active centers. Only 0.5 MPa of H_2_ was needed for the conversion of furfural, and the yield of CPO reached 90.2%.

#### 5.2.3. NiCo

The different performance of Ni, Co, and NiCo bimetallic catalysts was compared by using TiO_2_ as the support. Ni/TiO_2_ and Co/TiO_2_ mainly gave CPL as products, while 10%Co-10%Ni/TiO_2_ showed high selectivity to CPO (Table 3, Entry 8) [98]. A series of highly dispersed cobalt–nickel alloy nanoparticles (Co-Ni@NC) embedded in a porous nitrogen-containing carbon matrix have been synthesized through the pyrolysis of a metal–organic framework (MOF) template [99]. Cobalt and nickel showed strong interaction, and the introduction of N significantly improved the physical and chemical properties of the catalyst. The Lewis acid sites generated from metal oxides can both facilitate the generation of intermediate FOL and promote the FOL isomerization transformation.

#### 5.2.4. Other Bimetallic Catalyst

The promotion effect of cobalt, molybdenum, zinc, and nickel on Cu/CNT was systematically studied [96]. It was found that the addition of a second metal component to Cu/CNT had a significant impact on product distribution for the hydrogenation of furfural. CuZn/CNT was found to have the best performance for the production of CPO. Cu^0^ was considered as the active center, while the addition of Zn contributed to the fracture of carbon–oxygen bonds and the generation of carbon–carbon bonds. The excessive catalytic activity of Co could be adjusted by the addition of Fe to afford the Co-Fe alloy in a Co-Fe-Al prepared by the co-precipitation method. The introduction of Fe weakened the interaction between cobalt and Al_2_O_3_, promoted electron transfer between cobalt and iron, and inhibited the formation of B-acid centers. So, the side reactions for the formation of THFA or diols were suppressed. A 53.6% yield of CPO and a 37.9% yield of CPL were obtained (Table 3, Entry 13) [100].

**Table 3:** Conversion of furfural to CPO on bimetallic non-noble catalyst.

Entry	Catalyst	Concn. ^a^ (wt.%)	T (°C)	t (h)	P (MPa)	Conv. (%)	Yield ^b^ (%)	Ref.
1	NiCu/SBA-15	5	160	4	4	99.9	62	[57]
2	Cu-Ni-Al	5.3	140	8	4	100	95.8	[90]
3	Cu-Co-CP-500	1.9	170	1	2	>99	67	[95]
4	Cu-Co-OG-500	1.9	170	1	2	>99	(68)	[95]
5	CuNi_0.5_@C	5	130	5	5	99.3	96.9	[91]
6	Co@NCNTs	0.9	140	5	4	100	75.3	[101]
7	CuCo_0.8_@C-500	0.6	150	9	0.5	100	90.2	[97]
8	10%Co-10%Ni/TiO_2_	4.8	150	4	4	100	53.3 (16.3)	[98]
9	NiFe/SBA-15	5.7	160	2	3.4	99.8	90	[62]
10	Ni_2_Cu_1_/Al_2_O_3_	3.2	140	1	1	100	89.5	[94]
11	Co-Ni alloy	1.6	150	6	1.5	100	92.5	[99]
12	Cu-Ni/Al-MCM-41	1.2	160	5	2	99.0	96.7	[89]
13	1.5Co-1.5Fe-1.0Al	2.5	170	1	3	100	53.6 (37.9)	[100]
14	Ni_5_Cu_15_/m-SiO_2_	3.2	140	4	3	99.9	89.6	[92]
15	NiCu/SiO_2_-AE-450	3.2	150	6	2	100	95.4	[93]
16	CuZn/CNT	5.0	140	10	4	95	85	[96]

^a^ The concentration was calculated based on the data assuming the density of water is 1 g·mL^−1^. ^b^ The yield was the value provided by the literature or calculated by the product of selectivity and conversion. The value in the bracket represents cyclopentanol.

### 5.3. Single Noble Metal Catalyst

#### 5.3.1. Pd

Palladium is the widely used noble metal catalyst for the production of CPO from furfural. Hronec et al. [39] attempted to determine the effect of Pd/C when they first reported the conversion of furfural to CPO (Table 4, Entry 1). A 67.08% yield of CPO was obtained at 160 °C, and the selectivity to THFA increased at higher temperatures. To reduce the generation of THFA by-products and the polymerization of FOL, Bi could be used to partly poison Pd/SiO_2_ (Table 4, Entry 8) [102]. The continuous hydrogenation of furfural to produce THFA with FOL as the intermediate was dominant on Pd nanoparticles of Pd/SiO_2_. By segregating Pd nanoparticles with Bi, the formed FOL intermediate could not be quickly hydrogenated into THFA due to the lack of active Pd hydrogenation centers in the vicinity. The mechanism for the conversion of furfural to CPO and other products was studied in detail using Pd/C as the catalyst (Table 4, Entry 14) [103]. Through intermediate conversion and isotopic labeling experiments, the reaction pathway for the hydrogenation of furfural in hot water was revealed and summarized into four pathways. The role of water was also studied.

By comparing a series of different supports, carbon was found to be a support for Pd with higher activity for the hydrogenation of furfural. The selection of the carbon support had a significant impact on the products. Carbon-nanotube-supported Pd mainly gave CPO as the main product (Table 4, Entry 10) [104]. Alkaline support (MgAlO*x*) loading with Pd did not generate CPO. The effect of support for the Pd catalyst was also investigated by using different supports for Pd. The influencing factors of different supports on the reaction were studied. The Lewis acid site on the catalyst surface was beneficial to the formation of CPO, while the abundant presence of Bronsted acid generated a mixture of multiple products (Table 4, Entry 6) [105]. In order to avoid the adverse effects of Bronsted acid, MIL-MOFs with pure Lewis acid sites were used as the support for Pd (Table 4, Entry 9) [106]. High selectivity to FOL was obtained due to the absence of Bronsted acid. Fe-MIL-100-supported Pd with a higher dispersion of Pd had the highest activity compared to Fe-MIL-101 and Cr-MIL-101. The Fe-containing MOFs showed higher selectivity to CPO than Cr due to the higher oxophilicity. The Zr-MOFs (UiO-66) with Lewis acidity proved to be a good support for Pd for this transformation (Table 4, Entry 18) [107]. The introduction of nitro to the carboxylic acid ligand would further enhance the Lewis acidity on UiO-66-NO_2_. So, Pd/UiO-66-NO_2_ showed better results than Pd/UiO-66 in the conversion of furfural to CPO. The Lewis acidic molecular sieve was advantageous for this transformation as a support for Pd. Dai et al. [108] employed H-ZSM-5 as the support for Pd to catalyze the hydrogenation of furfural to CPO in water (Table 4, Entry 19). The effects of the palladium state, the Si/Al ratio in the zeolite, and the reaction parameters were investigated. The Brønsted acid sites in H-ZSM-5 were conducive to the hydrogenation of furfural to FOL, while the L-acid sites were beneficial for the isomerization of FOL. Pyrochlore in the form of A_2_B_2_O_7_ with pure Lewis acid proved to be effective for the conversion of furfural to CPO (Table 4, Entry 12) [109]. The acidity and oxygen vacancies were important factors affecting the transformation of furfural to CPO, which could be regulated by selecting different chemical elements while keeping the crystal structure of A_2_B_2_O_7_. The catalyst showed good stability, and a 95.0% yield of CPO was achieved on Pd/Y_2_(Sn_0.7_Ce_0.3_)_2_O_7-δ_. In order to achieve a better catalytic effect, pyrochlore supported on Al_2_O_3_ was developed. The addition of Al_2_O_3_ increased the surface area of the catalyst and increased the density of the oxygen defect by the in situ insertion of Al into pyrochlore (Table 4, Entry 16) [110]. The O defect selectively adsorbed C-O bonds and generated a large number of Lewis acid sites. La_2_B_2_O_7_ with different structures, where the B could be titanium, zirconium, or cerium, was used for the support for Pd (Table 4, Entry 17) [111]. Pd/La_2_Ti_2_O_7_ mainly produced CPO while THFA was mainly obtained on Pd/La_2_Ce_2_O_7._ The difference in the Lewis acidity and the interaction between the Pd and support was ascribed to this huge difference.

The physical separation of oxygen holes, Pd catalytic centers, and acid centers through the construction of hierarchical structures is a good choice to fabricate Pd catalysts for the hydrogenation of furfural to CPO. By first loading Pd onto cerium oxide, followed by overall loading onto acidic silicon oxide, the hierarchical structures were successfully constructed. The distance of Pd and acid sites could be well regulated to find the best distance, and the enriched oxygen vacancies promoted the hydrogenation of C=O. Through this synergistic effect of Pd, acid site, and oxygen vacancies, 84% selectivity to CPO was obtained from furfural (Table 4, Entry 20) [112].

#### 5.3.2. Pt

Pt/C showed the best performance among the common hydrogenation catalysts when the conversion of furfural to CPO was first reported. A 76.50% yield of CPO was obtained at 160 °C [39]. The porous carbon derived from bamboo shoots, doped with heteroatoms, was used as the support for Pt to produce Pt/C for the conversion of furfural to CPO. With a hierarchical porous structure, high content of nitrogen and oxygen functionalities, highly dispersed Pt nanoparticles, good water dispersibility, and reaction stability, Pt/C showed good activity for the hydrogenation of furfural, and the selectivity to FOL or CPO could be adjusted by the reaction conditions. At lower temperatures, FOL was the main product, while at higher temperatures, CPO was dominant (Table 4, Entry 4) [113].

The effect of the surface properties of TiO_2_ and the content of Pt on a Pt/C catalyst for the conversion of furfural to CPO was studied. The content of Pt mainly influences the activity, while the structure and properties of TiO_2_ and the Pt–TiO_2_ interaction had a great influence on the product distribution. Pt supported on the nanorod showed the highest selectivity to CPO (Table 4, Entry 15) [114].

#### 5.3.3. Au

The Au catalyst had weak catalytic hydrogenation ability, which could selectively catalyze the hydrogenation of aldehyde in furfural to form FOL without producing other side reactions such as furan ring hydrogenation. Therefore, it was suitable for the conversion of furfural to CPO. The best catalytic effect for the reaction of cyclopentanone preparation from furfural was found to be the Au catalyst loaded on titanium oxide among different supports, including metal oxides and acidic molecular sieves. After the optimization of the preparation conditions and loading of Au, furfural could be almost quantitatively transformed to CPO (Table 4, Entry 3) [115].

#### 5.3.4. Ru

The combination of acid center and Ru was a preferable catalytic system for the production of CPO from furfural. Li et al. used MOFs with acidic sites (MIL-101) as the support for Ru. Ru/C only had 40% selectivity for CPO with 46% selectivity for FOL due to the lack of acid sites. Ru/MIL-101 showed a 96% yield of CPO with negligible selectivity to FOL indicating the importance of the acid center in catalyzing FOL isomerization (Table 4, Entry 2) [116]. The introduction of additional solid acid additives to Ru/C to provide a furfuryl alcohol isomeric acidic center was a good choice for the high yield of CPO. An 81% yield of CPO could be obtained by the combination of Al_11.6_PO_23.7_ and Ru/C (Table 4, Entry 7) [117]. The selectivity to CPO or CPL could also be adjusted by simply changing the H_2_ pressure.

**Table 4:** Conversion of furfural to CPO on Pd, Au, Pt, and Ru.

Entry	Catalyst	Concn. ^a^ (wt.%)	T (°C)	t (h)	P (MPa)	Conv. (%)	Yield ^b^ (%)	Ref.
1	5% Pt/C	4.8	160	0.5	8	100	76.5	[39]
2	3 wt% Ru/MIL-101	9.0	160	2.5	4	>99	96	[116]
3	Au/TiO_2_-A	4.8	160	4	4	>99	99	[115]
4	Pt/NC-BS-800	2.3	150	4	3	>99	76	[113]
5	Ru/CNTs	1.1	160	3	1	99	87.4	[118]
6	4%Pd/f-SiO_2_	5	165	5	500 (psi)	98	87.2	[105]
7	Pt/C + Al_11.6_PO_23.7_	3.7	160	8	4	90	81.0	[117]
8	Pd-Bi/SiO_2_	4.8	150	2.3	5	-	57	[102]
9	Pd/Fe-MIL-100	2.4	150	6	4	99	92.2	[106]
10	Pd/CNTs	5	150	1	3	98.0	48.4	[104]
11	Pd/TiO_2_	2.4	170	4	2	>99	55.5	[119]
12	Pd/Y_2_(Sn_0.7_Ce_0.3_)_2_O_7-δ_	2.4	150	6	4	99.9	95.0	[109]
13	Pd/CNTs	4.4	150	1	3	>99	43	[103]
14	Pd/Cu-BTC	2.4	150	6	4	96.4	93.0	[120]
15	Pt/TiO_2_	2.5	170	2	2	98	49.4	[114]
16	Y_2_(Sn_0.65_Al_0.35_)_2_O_7-δ_/Al_2_O_3_	2.3	150	6	4	99.9	98.1	[110]
17	Pd/La_2_Ti_2_O7	2.4	150	6	4	99.5	82	[111]
18	Pd/UiO-66-NO_2_	1.0	150	5	1	98.9	95.5	[107]
19	2% Pd/H–ZSM–5(25)	2.3	160		3	93.7	91.8	[108]
20	Pd/CeO_2_/SiO_2_-10	2.5	150	3	2	93	78.1	[112]

^a^ The concentration was calculated based on the data assuming the density of water is 1 g·mL^−1^. ^b^ The yield was the value provided by the literature or calculated by the product of selectivity and conversion. The value in the bracket represents cyclopentanol.

### 5.4. Bimetallic Noble-Containing Catalyst

The addition of a second metal component in the noble metal catalyst can not only adjust the hydrogenation ability of the catalyst to avoid excessive hydrogenation of furfural, but also stabilize the noble metal nanoparticles and reduce the amount of noble metal. Carbon-supported Pd-Cu/C can be prepared by several methods, and its catalytic performance was tested by the conversion of furfural to CPO. The Pd-Cu bimetallic catalyst prepared by the chemical copper plating process under the presence of alcoholate–carboxylate ligands showed the best results. The main Cu species was Cu^+^ in the Pd-Cu catalyst, and the distribution of Pd^0^ and Cu^+^ determined the activity of the catalyst (Table 5, Entry 1) [121]. Deng et al. fabricated a bimetal Pd catalyst supported on cyanide with pure Lewis acid sites. The property of the acid sites could be fine-tuned by the selection of a proper second metal. The highest CPO yield was obtained on Pd/FeZn-DMC with moderate Lewis acidity, while the weak Lewis acidity on Pd/FeNi-DMC and Pd/FeCo-DMC made the main product of FOL. By in situ synthesis and subsequent reduction, Pd-Co nanoparticles were successfully introduced into the pores of UiO-66. Compared with the UiO-66-supported single-metal Pd, the introduction of a small amount of cobalt enhanced the activity while inhibiting the hydrogenation of the furan ring in FOL. Therefore, a high yield of CPO was obtained (Table 5, Entry 5) [122]. The water-induced hydrogen spillover in the interface of Pd and NiMoO_4_ could give sufficient Lewis acidity for the rearrangement of FOL (Table 5, Entries 6 and 7) [123]. As a result, Pd/NiMoO_4_ showed good selectivity to CPO or CPL. The selectivity to CPO or CPL could be controlled by the different precursors.

The bimetallic catalyst composed of Pt and Ru was also studied. The effect of Co on Pt was invaginated by varying the mole ratios of Pt to Co using carbon as the support (Table 5, Entry 2) [124]. The addition of Co to Pt highly increased the performance for the hydrogenation of furfural. There was about a 70 times increase in achieving higher yields of CPO compared to the monometallic Pt catalyst. Mo was used to modify Ru to form a carbon nanotube-supported bimetal RuMo/CNT catalyst (Table 5, Entry 4) [125]. It was found that the reduction temperature had a great influence on the properties of Mo species. Ru/CNT gave 81% selectivity of THFA, showing the strong furan ring hydrogenation performance. After the introduction of Mo, the hydrogenation reaction of the furan ring was completely inhibited, and a 74.3% yield of CPL was obtained under optimal conditions.

**Table 5:** Conversion of furfural to CPO on bimetallic noble catalyst.

Entry	Catalyst	Concn. ^a^ (wt.%)	T (°C)	t (h)	P (MPa)	Conv. (%)	Yield ^b^ (%)	Ref.
1	5% Pd + 10%Cu/C	4.8	160	1	3	98	92.1	[121]
2	Pt(3)Co(3)/C	1	180	5	1	100	75	[124]
3	Pd/FeZn-15	2.4	150	6	4	99.9	96.5	[126]
4	RuMo/CNT	4.8	160	4	4	100	9 (74)	[125]
5	Pd-Co@UiO-66	0.9	120	12	3	99	95.0	[122]
6	Pd/NiMoO_4_-Cl	2.4	150	6	4	>99	85.3	[123]
7	Pd-NiMoO_4_-AC	2.4	150	6	4	>99	(85.2)	[123]

^a^ The concentration was calculated based on the data assuming the density of water is 1 g·mL^−1^. ^b^ The yield was the value provided by the literature or calculated by the product of selectivity and conversion. The value in the bracket represents cyclopentanol.

## 6. Production of HCPN from HMF

### 6.1. Noble Metal Catalyst

Nb_2_O_5_-supported noble metal catalysts were first used for the conversion of HMF to HCPN by Ohyama et al. (Table 6, Entries 1–4) [40]. After comparing Au, Pd, Pt, and Ru loaded on different supports, Au/Nb_2_O_5_ was found to have the best results for the conversion of HMF to HCPN. The combination of the Au hydrogenation center and the Nb_2_O_5_ Lewis acid center achieved an 86% yield of HCPN from HMF. Furthermore, 28%, 43%, and 66% yields of HCPN were obtained on Pt/Nb_2_O_5_, Pd/Nb_2_O_5_, and Ru/Nb_2_O_5_, respectively. Then, the same group studied the combination of acidic metal oxides and Pt/SiO_2_ for this transformation (Table 6, Entry 5) [127]. It was found that acidic oxidants could greatly improve selectivity to HCPN, while alkaline or non-oxidized ones were not conducive to the generation of HCPN. Subsequently, a combination of Pt/SiO_2_ and lanthanide oxide was used for the transformation of HMF to HCPN. System research has been conducted on the influence of hydrogen ion concentration and oxide surface acidity (Table 6, Entry 6) [128].

It has been proved that MOFs with Lewis acid sites have suitable properties for the hydrogenation of furfural to CPO. The catalyst was also used for the production of HCPN. The differences in the application of Cu base MOF (Cu_3_(BTC)_2_ and FeCu-DMC) were compared by supported Pd nanoparticles. Pd/FeCu-DMC only offered BHMF as the product due to the weak acidity. However, Pd/Cu-BTC gave 90.9% selectivity to HPCN from the strong acidity of Cu-BTC. The higher dispersion of Pd on Cu-BTC made it have higher activity than Pd/FeCu-DMC (Table 6, Entry 9) [120]. Pd, Ru, Pt, and Au supported on MIL-MOFs were tested for the transformation of HMF to HCPN [120]. It was found that the Pd catalyst was more active than the others and had a higher selectivity to HCPN. Fe-MIL-100 proved to have the best results.

### 6.2. Non-Noble Metal Catalyst

Al_2_O_3_-supported Ni, Co, and Cu catalysts prepared through the co-precipitation method were used for the conversion of HMF in water (Table 6, Entries 7 and 8) [129]. Under the catalysis of the Ni-Al_2_O_3_ and Cu-Al_2_O_3_ catalyst, HCPN was mainly generated, while Co-Al_2_O_3_ produced 3-(hydroxymethyl)cyclopentanol (HCPL) as the main products. In addition to HCPN, 19% selectivity to 2,5-dihydroxymethyl tetrahydrofurfuryl alcohol (THFDM) and 15% of HCPL was also obtained on Ni-Al_2_O_3_. Cu had high selectivity to HCPN without THFDM and 1% of HCPL. To overcome the drawback of Ni-Al_2_O_3_, Fe could be used as the second metal component to increase the yield of HCPN (Table 6, Entry 14) [130]. The introduction of iron had a synergistic effect of forming the nickel-iron alloy, which improved the H_2_ adsorption capacity and adjusted the acidity and alkalinity of the catalyst. Xu et al. [131] obtained a 70.3% yield of HCPN from HMF catalyzed by the nickel–copper catalyst derived from the pyrolysis of Ni-Cu-MOF-74 (Table 6, Entry 12). Except for the role of solvent and reactant, the water could afford the necessary acid center for the rearrangement of BHMF, confirmed by the isotopic experiments employing heavy-oxygen water. Partially covering the NiCo alloy with a thin layer of carbon proved to be a good catalyst for the hydrogenation of HMF to HCPN (Table 6, Entry 16) [132]. Compared to the monometallic counterpart (Ni@C and Co@C), Ni_0.5_Co_0.5_@C led significantly in both activity and selectivity. In addition, the (3-aminocyclopentyl)methanol could be obtained on the same catalyst in a one-pot cascade way.

**Table 6:** Conversion of HMF to HCPN on metal catalyst.

Entry	Catalyst	Concn. ^a^ (wt.%)	T (°C)	t (h)	P (MPa)	Conv. (%)	Yield ^b^ (%)	Ref.
1	Au/Nb_2_O_5_	0.8	140	12	8	>99	86	[40]
2	Pt/Nb_2_O_5_	0.8	140	12	8	>99	28	[40]
3	Pd/Nb_2_O_5_	0.8	140	12	8	>99	43	[40]
4	Ru/Nb_2_O_5_	0.8	140	12	8	>99	66	[40]
5	Pt/SiO_2_ + Ta_2_O_5_	0.8	140	12	3	100	82	[127]
6	Pt/SiO_2_ + Nd_2_O_3_	0.8	140	30	3	100	88	[128]
7	Cu-Al_2_O_3_	0.5	180	6	2	100	86	[129]
8	Co-Al_2_O_3_	0.5	140	48	2	100	(94)	[129]
9	Pd/Cu-BTC	3.2	150	24	4	99.5	90.4	[120]
10	Pd/Fe-MIL-100	3.2	150	24	4	99.9	85.4	[106]
11	Pd/FeZn-15	3.2	150	24	4	99.9	87.4	[126]
12	Ni-Cu/C	4.8	140	5	2	>99	70.3	[131]
13	Pd/Y_2_(Sn_0.7_Ce_0.3_)_2_O_7-δ_	3.2	150	12	4	99.9	92.4	[109]
14	Ni-Fe/Al_2_O_3_	2.1	160	4	4	100	86	[130]
15	Pd/La_2_Ti_2_O7	3.2	150	6	4	99.8	82.3	[111]
16	Ni_0.5_Co_0.5_@C	1.2	140	7	2	99	92	[132]

^a^ The concentration was calculated based on the data assuming the density of water is 1 g·mL^−1^. ^b^ The yield was the value provided by the literature or calculated by the product of selectivity and conversion. The value in the bracket represents cyclopentanol derives.

## 7. Conclusions

The synthesis of cyclopentanone derivatives from furfural and HMF is an excellent example of the efficient utilization of renewable biomass resources for chemical production. The transformation of the furan ring to the carbon ring mainly occurred through the Piancatelli rearrangement reaction. The transformation requires a series of hydrogenation-rearrangement steps catalyzed by metal hydrogenation catalysts in acidic aqueous solution. Water is an indispensable reactant in the transformation process and is usually an ideal solvent for the reaction. To achieve the isomerization of the molecular skeleton, the reaction needs to be carried out at a high temperature. Metal catalysts play a core role in the reaction. Both non-noble metals, Cu, Ni, and Co, and noble metals Pd, Au, Pt, and Ru, can effectively catalyze the reaction.

(1) Non-noble metals Cu, Ni, and Co showed different catalytic performances in the reaction. Cu has high selectivity for the reaction and generates no furan ring hydrogenation and carbon–oxygen bond cleavage by-products, but the catalytic activity of Cu for the reaction was low. Ni and Co had higher activity but were prone to furan ring hydrogenation and other side reactions. Moreover, Co was difficult to stay at the cyclopentanone derivative stage, and the products were usually further hydrogenated to form cyclopentanol derivatives.
(2) Since the furan rearrangement reaction requires a high temperature, high temperature is still needed for noble metal catalysts. The most studied catalyst for this reaction is the noble metal Pd. Pd catalysts have high catalytic hydrogenation activity for the aldehyde groups in HMF, and furfural and can easily obtain cyclopentanone derivatives with high yields. However, Pd catalysts still need to be carefully designed to avoid the production of furan ring hydrogenation products. Compared with Pd, Au catalysts have lower activity, but are less sensitive to furan ring hydrogenation and carbon–oxygen bond cleavage and can easily obtain higher yields of cyclopentanone derivatives.
(3) Bimetallic catalysts combine the advantages of different metal catalytic centers, suppress unfavorable factors, and obtain higher catalytic performance than monometallic catalysts by adjusting the electronic structure of the active center through the interaction between the metals. On the other hand, the addition of a second metal component can reduce the amount of noble metal used and is conducive to reducing the reaction cost.
(4) The support plays an important role in the metal hydrogenation of catalysts. In addition to being able to disperse and stabilize the metal active centers, more importantly, it can provide the acidic sites required for the rearrangement reaction. The Lewis acid site is more favorable for the reaction than the Bronsted acid.

## 8. Prospection

Since the preparation of cyclopentanones by furfural and HMF was reported, it has attracted wide interest due to its wide application. Up to now, many catalytic systems for the reaction have been developed. Some of the catalytic systems can achieve a cyclopentanone yield as the stoichiometric reaction. Further in-depth research needs to be carried out in the following areas in future research.

(1) The role of each component of the catalyst in the furfural and HMF conversion reaction needs to be further studied in detail. What other roles does the catalytic hydrogenation metal active center have in addition to providing the hydrogenation site? Different hydrogenation centers often have great selectivity differences in addition to the different hydrogenation activities. It indicates that the metal catalyst still has other roles in the reaction process. Uncovering these roles will guide the design of high-performance catalysts. Recent studies have found some factors, such as the generation of acidic sites through the hydrogen spillover effect.
(2) The Piancatelli rearrangement, a key step of the furfural-prepared cyclopentanone derivative reaction, is a ring opening followed by the electrocyclic reaction. Combining traditional thermal catalysis with microwave-assisted reactions, photocatalysis/electrocatalysis, etc., may promote the rearrangement reaction at lower temperatures, thus inhibiting the occurrence of side reactions and obtaining cyclopentanone more efficiently.
(3) The concentration of reactants is one of the crucial indicators for industrial applications. Most of the catalytic systems reported up to now have a reactant concentration of no more than 5%. Few studies have been conducted under high-concentration conditions. Under high-concentration conditions, it is necessary to accurately design the catalyst to ensure catalytic activity and avoid catalyst deactivation, and more attention should be paid to selective regulation. In addition, it is necessary to solve the problem that intermediate products are more prone to aggregation under high-concentration conditions.

## Figures and Tables

**Table 1:** Catalytic performance of NiFe/SBA-15 catalyst in different solvents.

Entry	Volume Ratio (Methanol/Water)	Conv. (%)	Selectivity (%)		
			FOL	THFA	CPO
1	100/0	99.8	88.3	1.7	0
2	75/25	99.8	1.9	95.8	2.3
3	50/50	79.9	58.7	17.2	24.1
4	25/75	67.8	27.8	13.5	58.6
5	0/100	53.5	12.5	4.3	75.6

Adapted with permission from Ref. [62]. Copyright 2019 American Chemical Society.

## Data Availability

Not applicable.

## Abbreviation

CPL	cyclopentanol
CPO	cyclopentanone
FOL	furfuryl alcohol
HCPL	3-(hydroxymethyl)cyclopentanol
HCPN	3-hydroxymethylcyclopentanone
HMF	5-hydroxymethylfurfural
MOF	metal–organic framework
THFA	tetrahydrofurfuryl alcohol
THFDM	2,5-dihydroxymethyl tetrahydrofurfuryl alcohol
